# Community-led responses to COVID-19 within Gypsy and Traveller communities in England: A participatory qualitative research study

**DOI:** 10.1016/j.ssmqr.2023.100280

**Published:** 2023-06

**Authors:** Alicia Renedo, Rachel Stuart, Charlotte Kühlbrandt, Pippa Grenfell, Catherine R. McGowan, Sam Miles, Serena Farrow, Cicely Marston

**Affiliations:** a Faculty of Public Health & Policy, London School of Hygiene & Tropical Medicine, 15-17 Tavistock Place, London, WC1H 9SH, UK; b College of Business, Arts and Social Sciences, Brunel University London, Kingston Lane, Middlesex, UB8 3PH, UK

**Keywords:** England, Gypsy, infection control, pandemic, Travellers, COVID-19

## Abstract

Individuals were asked to play an active role in infection control in the COVID-19 pandemic. Yet while government messages emphasised taking responsibility for the public good (e.g. to protect the National Health Service), they appeared to overlook social, economic and political factors affecting the ways that people were able to respond.

We co-produced participatory qualitative research with members of Gypsy and Traveller communities in England between October 2021 and February 2022 to explore how they had responded to COVID-19, its containment (test, trace, isolate) and the contextual factors affecting COVID-19 risks and responses within the communities.

Gypsies and Travellers reported experiencing poor treatment from health services, police harassment, surveillance, and constrained living conditions. For these communities, claiming the right to health in an emergency required them to rely on community networks and resources.

They organised collective actions to contain COVID-19 in the face of this ongoing marginalisation, such as using free government COVID-19 tests to support self-designed protective measures including community-facilitated testing and community-led contact tracing. This helped keep families and others safe while minimising engagement with formal institutions.

In future emergencies, communities must be given better material, political and technical support to help them to design and implement effective community-led solutions, particularly where government institutions are untrusted or untrustworthy.

## Highlights

• Co-produced qualitative research with Gypsy and Traveller communities in England.
• Gypsies and Travellers organised collective actions to contain COVID-19.
• Community-led solutions included community-facilitated testing and contact tracing.
• Community responses developed in the face of marginalisation and discrimination.

## 1 Introduction

Public health measures have important social dimensions that affect intervention designs, implementation, acceptability, compliance, and ultimately effectiveness for any population. Ignoring these social factors has hindered programmes both in the past, and during the COVID-19 pandemic (Marston, Renedo, & Miles, 2020, McGowan et al., 2020). In this paper we discuss how Gypsies and Travellers worked to contain COVID-19 in communities in the face of chronic and ongoing discrimination and marginalisation.

Individuals were urged to play an active role in COVID-19 infection control. In the UK, government rhetoric emphasised personal sacrifice and responsibility for the public good (Andreouli & Brice, 2022), largely ignoring the “social, economic, and political vulnerabilities” that can affect how people engage with public health measures such as COVID-19 testing (Bevan, Stage Baxter, Stagg, & Street, 2021, p.23). Individuals will engage or not engage with infection prevention and control measures depending on practical issues such as accessibility, and broader experiences and histories such as their interactions with health services and government institutions, their living conditions, and wider socio-cultural, material, and political needs. COVID-19 testing - a measure at the core of the global public health response to the pandemic-needed to be considered as “embedded in people's everyday routines, livelihoods and relationships”, and engagement with it as potentially influenced by different types of vulnerabilities (e.g. social, economic, health, political) (Bevan et al., 2021, p. 1).

Gypsy, Roma and Traveller communities (sometimes combined under the initialism GRT to refer to diverse groups of people with some commonalities based on nomadic lifestyles, ancestry or culture (James, 2015)) are particularly affected by intersecting vulnerabilities and hostilities (James, 2020; United Nations, 2016). The COVID-19 pandemic exacerbated existing health disparities (Nazroo & Becares, 2020; Platt & Warwick, 2020). GRT communities in Europe experience barriers to healthcare access (McFadden et al., 2018; Office for National Statistics (ONS), 2022b), as well as experiencing discrimination and hostility from healthcare providers (McFadden et al., 2018) and from wider society (James, 2015). A range of systemic factors impede access to and engagement with healthcare for individuals from GRT communities globally (McFadden et al., 2018), including “health service issues” (p.78) (e.g. registration requirements such as having a fixed/permanent address, staff reluctance or unwillingness to visit sites); discriminatory treatment by healthcare staff (e.g. “… hostile, patronising, judgemental, unsympathetic and even abusive attitudes” (McFadden et al., 2018, p. 78)); health literacy and language barriers; lack of cultural awareness among healthcare staff; and mistrust of health services based on poor past personal or others’ experiences (McFadden et al., 2018).

Wide-ranging and intersecting inequalities and challenges faced by Gypsy and Traveller communities in Britain (England, Scotland and Wales) include (Cemlyn, Greenfields, Burnett, Matthews, & Whitwell, 2009): high levels of poverty; low employment rates; “children's educational achievements are worse […] participation in secondary education is extremely low” (p. v); “high suicide rates” (p. vi); “accelerated criminalisation at a young age, leading rapidly to custody” (p. vi); “little or no recognition” of culture and identity (p. vi); and “lack of access to culturally appropriate support services for people in the most vulnerable situations” (Cemlyn et al., 2009, p. vi). Gypsy and Traveller communities in the UK experience lack of secure, culturally appropriate and adequate accommodation (Cemlyn et al., 2009; James & Southern, 2019). This can push people into sub-standard living conditions that threaten health and wellbeign (e.g. sites with deteriorated water and sewage fittings) (Cemlyn et al., 2009; James & Southern, 2019; Millan & Smith, 2019). Those who buy land and live in private sites can encounter racist treatment and barriers to planning applications when trying to develop their sites (Cemlyn et al., 2009).

We conducted this study in England, where the challenges Gypsy and Traveller communities experience were being exacerbated by the Police, Crime, Sentencing and Courts Act 2022, which was passing into legislation while this research was being conducted. The Act makes residing in a vehicle on private or public land without authorisation a criminal offence (Burrows, Green, Speed, & Thompson, 2021) and carries a sentencing tariff that includes a large fine, up to three months of imprisonment and repossession of caravans. The Act is likely to have a profound impact on the lives of Gypsy and Traveller communities who follow a nomadic lifestyle.

It was in this challenging context that Gypsy and Traveller communities experienced the COVID-19 pandemic, and were asked to test, trace contacts, and self-isolate to help reduce the impact of the virus.

## 2 Testing, biopolitics and citizens’ role in virus containment

The UK government developed a centralised COVID-19 test and trace mechanism and provided two testing methods: Real-time reverse transcription polymerase chain reaction (PCR) and rapid antigen lateral flow device (LFD) tests. Both tests were free of charge at point of use. LFDs did not require swabs to be taken to laboratories and could produce quick results at home. Rapid access to test results was important for timely self-isolation and speedy reporting - crucial for effective contact tracing and reducing onward infection (Crozier, Rajan, Buchan, & McKee, 2021). Infection control also depends on people engaging with testing (Crozier et al., 2021) and contact tracing mechanisms, as well as adhering to adequate self-management behaviours (e.g. self-isolating after a positive test) (Street & Kelly, 2021).

COVID-19 testing could be seen as part of wider bio-political mechanisms through which governments introduce instruments and codes of practices to govern people's everyday life, making the individual responsible for reducing infection (Jayasinghe, Jayasinghe, Wijethilake, & Adhikari, 2021). In the UK, government rhetoric emphasised individual responsibility and the self-governing efforts of individuals to help mitigate the risks of COVID-19 infection (e.g. social distancing, restriction on movements and social gatherings) (Andreouli & Brice, 2022; Jayasinghe et al., 2021). UK government messaging framed COVID-19 containment (test, trace, self-isolate) as a person's moral duty to others; a way for “individuals to manage their own risk and the risk to others“ (UK Government UKHSA, 2021). Individual citizens ought to test “in the name of life and health” (Rabinow & Rose, 2006, p. 195) to protect themselves and the population.

The focus on individual responsibility for virus containment, however, risks ignoring the social and structural dimensions of people's lives and presupposes that individuals are free to behave in particular ways and be able to engage with public health systems (Maunula, 2013). For instance, the impact of testing mechanisms will be limited if individuals are not supported to self-isolate if they test positive (Crozier, McKee, & Rajan, 2020) and to seek tests in the first instance. Past experiences of mistreatment by healthcare providers and other authorities also need to be considered as these may deter people from engaging with infection control mechanisms (McGowan et al., 2020).

The costs to individuals engaging in testing should also be considered, such as self-isolation on positive test, time invested in frequent testing, and loss of income while self-isolating (Bevan et al., 2021).

## 3 Methods

We were commissioned in 2021 by the UK Department of Health and Social Care (DHSC) via the National Institute for Health Research (NIHR) Policy Research Programme to conduct this work, to provide urgent information to inform policy. The project “Routes: New ways to talk about COVID-19 for better health” used a participatory qualitative approach. The overarching project was co-produced with members of Gypsy, Roma, and Traveller communities (GRT) and migrant workers in precarious jobs in England, and to some extent with DHSC officials (interviews included questions addressing policy priorities). Involvement of communities is crucial to co-produce inclusive solutions for emergency preparedness and response that are accepted by communities and work well to meet their health security needs (Marston et al., 2020). We co-produced the work throughout. We engaged in dialogues and collaboration with members of the communities and other stakeholders (e.g. Civil Society Organisation (CSO) staff, academic researchers, members of communities) to identify and refine research questions, including discussing whether or not this would be an appropriate project to engage in at all. We co-generated data, and co-produced the analysis and recommendations with communities. We trained and worked with co-researchers from communities who were involved in various aspects of the work – e.g. supporting recruitment, interviewing participants, interpreting findings. In this paper we focus on the experiences of Gypsy and Traveller communities, which were distinct from those of the other groups. Our analysis is informed by the full body of work.

Interviews were conducted by three team members with relevant backgrounds (authors RS, CK, SF). RS is an academic researcher from a Traveller background; SF is a co-researcher from a Romany Gypsy Showperson background; CK has worked extensively with Roma communities.

Collaborating and developing dialogue with communities throughout the project was crucial to ensure the knowledge generated via the research incorporated their voices and expertise. Members of marginalised communities are better placed to produce knowledge about their own experiences and realities than outsiders and bring this expertise into the research process (Collins, 1986, 2000). The analysis ultimately combines different types of expertise and knowledge(s) (i.e. academic and lived experience) Miles, Renedo, & Marston, 2018, Renedo, Komporozos-Athanasiou, & Marston, 2018.

As well as the informal conversations, dialogues and other participatory work, we conducted in-depth interviews with 47 individuals from GRT communities, of whom 30 were members of Gypsy and Traveller communities (the focus of this paper), between October 2021 and February 2022.

We aimed for diversity in our sample in terms of age and gender, type of living arrangements (e.g. private site, council-run site) and identity (Gypsy, Roma, Traveller). We conducted interviews in areas within four broad geographical locations in England (South East/East, North East including Yorkshire, South West, and West Midlands). Many participants self-identified into overlapping categories (e.g. Gypsy Traveller), highlighting the plural and fluid nature of identities within these communities (Condon et al., 2019).

Interview topics and questions were informed by discussions with community members. In the interviews we explored participants' experiences of the COVID-19 pandemic in the context of their lives and of COVID-19 public health responses (testing, self-isolation, vaccination). We adopted a holistic approach and included questions to understand the broader context of participants’ lives beyond COVID-19, including living environment, relationships with healthcare and other services more generally and, crucially, issues identified as important by communities that affect how they engage with public health interventions.

In this paper, we present findings from the interviews with Gypsies and Travellers, which were distinct from those of the Roma communities. We draw on data from qualitative in-depth interviews with 23 women and seven men from Gypsy and Traveller communities (12 aged 20–60 years old, 9 aged 30–39 years old, 6 aged 40–49 years old, and 3 aged 50–69 years old). In some cases, members of the research team and co-researchers used their existing contacts within communities to secure interviews. In locations where we were ‘outsiders’, we worked with trusted networks to enter these communities. We conducted nearly all our interviews in person in community sites (e.g. homes) and in some cases there was more than one participant in the interview, responding to participants' cultural practices.

Participants gave signed informed consent. Consent information was communicated verbally when necessary. We gave interviewees £40 to compensate for their time and travel costs, and provided referral information to health and support services. We audio recorded interviews, which were then transcribed verbatim by a transcription agency. We paid co-researchers a set rate based on NIHR guidelines and agreed in advance (National Institute for Health Research, 2022).

The study was approved by the London School of Hygiene & Tropical Medicine Research Ethics Committee (No. 26440).

The analysis followed some of the principles and practical steps of Charmaz’ constructionist grounded theory (e.g. open coding, memo-writing), using iterative methods of constant comparison that are particularly useful for exploring lived experience (Charmaz, 1990, 2006). The team did the analysis jointly. Different team members focused on different interviews, and met regularly for analytical discussion meetings. We developed some of the codes *a priori* to explore the thematic areas of interest of the test and trace journey and the wider public health response (e.g. booking and ordering tests, testing, reporting contacts, self-isolation, vaccination). Other codes and themes emerged inductively from the interview data. During the analysis we also drew on fieldnotes taken after interviews, on team analytical memos, and on team analytical discussion meetings. Through these analytical meetings we contextualised and refined the analysis, informed throughout by RS's lived experience of being from a Traveller background. As part of the participatory approach, we also discussed the findings with two people from Gypsy and Traveller communities (SF and another person) to enrich the analytical process.

Interviews are numbered. These numbers were allocated within the wider study from which the Gypsy Traveller interviews are drawn (Kühlbrandt et al., 2023, Marston et al., 2022).

## 4 Findings

Participants organised and led collective actions to protect their own and others’ health, and to navigate constraints of the public health response to the pandemic. They used free-of-charge COVID-19 public health services and resources (e.g. PCR testing, LFD testing) in tandem with their own protective measures such as community-led contact tracing, which evolved as mechanisms to keep their families and others safe and to support community needs during the pandemic. These protective measures were not without challenges, and were developed in the face of chronic and ongoing marginalisation, exclusion from health services, police harassment, surveillance, discrimination and constrained living conditions.

### 4.1 Communities’ experiences in context: policing, surveillance, poor treatment in healthcare services and difficult living conditions

#### 4.1.1 Policing and surveillance

Participants told us about different experiences of police harassment and surveillance by local authorities and neighbours, including policing during lockdowns (Quote 1). Some participants for instance lived in council-run traveller sites surrounded by CCTV cameras pointing at their homes/trailers; some sites were locked with council-installed barriers and padlocks, which residents could not open.

One man said he was harassed by the police and fined for breaching COVID-19 lockdown regulations while on his way to the police station, at police request:

“I’ve got a £400 Coronavirus fine from the courts, because when he got arrested […] they’ve [police] rung me up, and said, can you be his appropriate adult? […] And then they’ve ended up pulling me over [the police], they put the handcuffs on me, searched my car, and then give me a fine for, for breaching Coronavirus regulations.”

*Quote 1 (Interview 6)*

One woman told us that her son's death, prior to the pandemic, had not been adequately investigated by the police (Quote 2). In addition, when her husband had arrived at the hospital to see their son, he was arrested. The police also searched another family member's car. The woman told us how she tried to spend time with her son's body in the hospital, but the room was heavily policed, and her requests were disregarded – an example of the lack of cultural awareness by the police: sitting with the deceased is an important part of the grieving process for Travellers.

[…] my son laid there, and there was police officers round my son, and they kept, they kept saying to us, “you’ve got to leave the room now, you’ve got to leave the room” […] And um, I said to them, you know, “why, why have we got to leave the room? He’s laying here, you know, we just want to sit with him.” […] He [hospital staff] said, “you should allow her”, he said, “you sit there with your son”. And he, and one of the police officers, he actually said, “well no, I’ll sit with you.”

*Quote 2 (Interview 43)*

The experience of this family resonates with pre-COVID-19 experiences of author RS's family: on two separate occasions when family members died, other members of the family were arrested. This included the raiding of the homes of several family members within hours of a teenage boy being killed in a car crash.

Two participants also told us they had experienced hostile surveillance by neighbours who reported them to the police. One woman told us that during the pandemic a non-traveller neighbour had reported her to the police. The neighbour had taken a photo of her chatting to someone while buying an ice-cream from an ice-cream van, and sent it to the police, trying “*to make out it was a gathering*”. The council sent her a letter saying she had breached her contract with this “*antisocial behaviour*”. One woman said her neighbours had installed cameras overlooking her house. She described intense surveillance by social services and other authorities, and attempts to evict her, as a result of calls and racist assumptions made by her neighbours. She told us about her neighbours characterising her family as dirty and infectiou*s*, that they assumed she sold drugs and that she was being “*stalked*” by them.

#### 4.1.2 Healthcare experiences: poor treatment and disregard for people's voices

The government push for citizens to engage with public health responses to COVID-19, including vaccination (Kühlbrandt et al., 2023), contrasted with the healthcare neglect Gypsy and Traveller communities otherwise experience. Participants discussed past and ongoing experiences of poor care. They told us that during the pandemic health visitor services were reduced, as was special needs support for children. They said there was little follow-up and rehabilitation after operations. They also found it hard to access GPs, for instance one described a receptionist obstructing access to appointments. They talked about poor treatment from healthcare staff including having their concerns dismissed, receiving poor quality care at hospital (e.g. being neglected during a hospital stay), and having problems getting ambulances onto sites for urgent care – both because of council-installed barriers and, they said, because ambulances “*didn't really want to come in*”. Many participants were carers for the elderly, for newborns, or for children with special developmental support needs.

One woman whose baby was a few months old when the pandemic started told us she had struggled to get access to healthcare for the baby (e.g. GP appointments and check-ups). She felt isolated and lonely; health visitors never contacted her, and she contrasted this neglect with the “*big push about postnatal depression*”. She told us about an encounter with a healthcare professional who patronised and lectured her about what not to feed her baby, based on preconceptions about Gypsy women's weaning practices. She felt “*scrutinised and judged*” and was not given space to talk about her concerns (Quote 3):

“I’ve got a kid that’s like, you know, six months behind her percentile […]. But nobody, the only message I ever had, […] It was like a, one of the first reviews they did, and it was a very long lecture about not feeding her mashed potato at three month old. I think somebody’s told this woman that Gypsies feed their babies mashed potato […] she knew I was a Gypsy woman. And she just kept saying it. I was like, I will wean her when she’s ready, when, I’ve read the books […] And I plan on weaning her on vegetables that are not sweet […]. But she just kept going on about this mashed potato […] it’s like, excuse me, but I think you’ve got some very racist views.”

*Quote 3 (Interview 45)*

The same woman's baby had a routine 12-month checkup by phone. She said she worried that if something was wrong she would be blamed for neglect and this particularly worried her because of a “spike” in Traveller children being taken into care. She talked about discriminatory treatment against Travellers in health services (Quote 4):

“It's much easier just to disregard us, put us off. Give us ridiculous appointments and ridiculous conditions to those appointments, that you can’t keep. And the, the thing that really annoyed me is, if I hadn’t have rang back [the GP] and said, I can’t get a, a PCR test [that the GP requested for her daughter’s appointment], it would have been put down as though I’d missed an appointment."

*Quote 4 (Interview 45)*

Interviewees also described their concerns about vaccination being disregarded. One woman who acted as representative of the Gypsy and Traveller community at a health forum told us about being patronised and “*spoken to like an idiot*” by a doctor when asking whether the vaccine caused infertility. She said that the doctor did not address her concern and instead explained to her how women conceive.

#### 4.1.3 Living conditions

Certain characteristics of participants' living conditions (gated council sites with locked barriers, crowded sites with little space between caravans, no access to water) made health risks and marginalisation particularly visible to the communities forced to endure them. A participant spoke of her experience breaking the lock on the council site entrance for the ambulance to get her husband who was very unwell with COVID-19: “*We had to have a gate codes broke off, because they refused to give us the code.*” She wanted to move off the site because the council treat them like “*animals*”. A participant said they were not allowed to have the gate codes because “*they don't trust us*”. One woman was worried about not getting urgent care on time during the pandemic because years ago someone at the site died waiting to be attended by the ambulance. Instead the police came as if someone had “*committed a murder*”. The police thought the person had died (in a moment of panic the family had screamed he was dead) and held the ambulance off for 30 ​min. Others talked about ambulances and food delivery vans not wanting to come to the sites. One woman, very ill with COVID-19, had had to call an ambulance two or three times. She explained that the ambulance “*didn't really want to come in*” but “*they had no other choice*”.

Other infrastructure problems on sites also made it harder to keep safe and protect health during the pandemic, including overcrowded living conditions, problems getting post to sites with no postcode (e.g. GP and hospital letters) and council neglect: some had no access to water and electricity during lockdown. One woman with children living in a private site where a lot of vulnerable people lived told us about her efforts to follow official COVID-19 guidelines for handwashing after the local council had disconnected the water supply during a dispute about planning permission (Quote 5). Faced with the obstacle of not having access to running water, she had to devise her own intricate hygiene regime to disinfect her children's hands. She recalled “*being told*” to adhere to UK government hand-washing guidelines. Yet to adhere to such practice she had to resort to her own resources (buying antiseptic solution) and engage in a more demanding sanitising regime. Her disciplined hand-washing regime took a toll on the children's skin:

We were being told now to wash our hands, like, continuously during the day, that they, the safe thing to do was keep washing your hands. We didn’t have the water to do it […] the council refused us water […] with the handwashing, there were dishes of water, like I was going crazy because I had a bucket at the door with Dettol [concentrated antiseptic solution]. […] What I used to do is I’d keep a bucket of water at the door and pour a three cupful of Dettol in it and leave a towel on the bench beside, bench outside the caravan door […] and what I used to do was, and even when the kids were going out, dip your hands in the bucket, wipe, any time they were going back in, dip your hands in the bucket, wipe. They had no skin left, I had to go and buy hand cream in the end where it was going on and on for weeks and weeks. […]

*Quote 5 (Interview 35)*

### 4.2 Creating community responses: self-led solutions and community organising to protect self and others

Communities engaged with COVID-19 containment measures and organised collective action to keep their immediate and extended family circles safe and to avoid spreading the virus. They did this in the face of constrained living conditions and experiences of discrimination illustrated above. Free COVID-19 tests were widely used, with positive test results reported into personal networks to notify contacts and help stop the spread, and self-isolation was taken seriously. These protective measures were used in tandem with self-led and community organising solutions, which involved communities mobilising to provide mutual assistance, including; (1) helping with testing and contact tracing to keep families safe and avoid spreading the virus, and (2) helping with basic needs during self-isolation and lockdowns.

#### 4.2.1 Testing

Testing was facilitated by tests being available free of charge. Participants engaged in testing for predictable reasons such as having COVID-19 symptoms, compulsory testing for school/work/hospital visits or being a contact of a positive case. Testing and self-isolation were underpinned by desire to keep families safe and avoid spreading COVID-19 to the wider community. For example, a woman who was a key worker during lockdown was constantly worried about being infected and bringing the virus home to her parents (Quote 6). She tested at least once per week and chose rapid tests as soon as she heard about them, even though the test centre was further away. Having access to rapid tests meant she did not have to worry about the possibility of spreading COVID-19 to others while she was waiting for the results.

When you’ve done the [PCR] test and then you're waiting on the day or something, you think to yourself, oh God. Like what if I have got it, what if I haven't got it. So what if I need to do something and then I have got it and then I'm going around spreading it to someone, do you know what I mean? […] So it gives you that little bit of a thing where you got the, the results quicker and you haven't got the worry of …

*Quote 6 (Interview 44)*

A similar sense of responsibility towards others was shared by a woman who explained she did not “*massively over-test*” because she did not have symptoms but tested for “*security*” if she had been in contact with a positive case. She said testing was “*never about me, it was always about people around me*”. She also talked about how community-led assistance to support testing of site residents developed in response to lack of official support. No officials or healthcare professionals had come to her site to give information about how to access tests (Quote 7). One of the younger residents at the site worked out how to book appointments for PCR tests and explained the process to the residents, supporting them with the bookings and explaining what to expect would happen at the testing centre.

[…] we didn’t have a liaison officer to come down and say, well, that’s what you need to do [for booking a test], that’s where you need to go. But once, once one of the younger girls that was there found the process of how to do it and kind of showed us all one by one of, well, you go on that app, and you do that, and then you go to there, and then you do it through the bag, and you give it out through the car window back to the people and things.

*Quote 7 (Interview 35)*

This participant also told us about residents at her site being forgotten by the council: site residents had not been informed of an outbreak in a nearby town and that they needed to get tested. Everyone in town had been asked to get tested at a particular centre*: “[they] didn't come to us, so what, we were OK to die then were we, OK, to get it and die*?”

Early in the pandemic some participants had found it difficult to access tests, for instance, finding it hard to book tests. Some did not know how and where to access tests and would have welcomed more information. Access to PCR and LFD tests was facilitated by the community: community and family members collected and distributed LFDs, and helped others with booking tests and accessing testing centres. During our engagement with communities as part of our participatory work we were told one organisation had produced videos with instructions about testing for members of GRT communities and circulated these via their networks. One woman told us about working out a way to navigate limits of the test ordering system to procure tests for others in the community:

I know, you, you’re not supposed to, are you? [obtain LFD test for others] Because I think when they send them out like you can only use like one email address, but I’ve got like four or five email addresses. And some people up here, they’re like, I can’t, it won’t let me order them or I can’t figure it out. So, I just order it in their name, but with my email addresses. So that other people up here can get them as well […] Or I’ve given like boxes of them to people before.

*Quote 8 (Interview 20)*

One participant, who lived in a house rather than on a Traveller site, told us she had never been tested for COVID-19 because "*it's all fake*” but had taken her son for a COVID-19 test after he was exposed to a positive case at school. She talked about COVID vaccination as a government tool to monitor people. She also said that she did not test because she was constantly under stress from her neighbours: their constant surveillance, accusations, and racism.

#### 4.2.2 Self-isolation, contact tracing and other forms of social protection

All participants who had been infected said they had self-isolated and often described making a great deal of effort to avoid infecting others. For example, one participant told us she wore a mask at home to protect her partner and children and carried out repeat LFD testing during and at the end of the isolation period to double check they were all negative before they went out. The family waited a further four days before visiting vulnerable relatives. Another woman had symptoms so booked a PCR test (which was positive) via drive-through to avoid infecting others.

For some, self-isolation was very difficult to achieve because of crowded living conditions, lack of financial support, or lack of childcare. It involved personal and financial sacrifices. One woman living in a caravan with one bedroom with very little space to self-isolate, said three of her children and her partner had had to self-isolate in one room (Quote 9). She also spoke about the financial costs of having to take time off from work for repeated periods of self-isolation when members of her family became ill, either simultaneously or consecutively.

“I had four at one time, all tested positive for Covid, so they were all shut in that room there. […] And literally, the room’s no bigger than you could swing a cat in”.

*Quote 9 (Interview 27)*

Participants universally told us that they would report any positive COVID-19 test result directly to contacts through their personal and social networks. There was no evidence of stigma associated with being infected: participants wanted to tell others to protect them and help stop the spread. There was a shared sense of responsibility towards others and to avoid being a risk to others.

Community-led solutions for testing and contact tracing involved reporting positive status to the community and community contact tracing (notifying contacts). Communities self-organised to warn each other of positive COVID-19 cases and contacts via phone, WhatsApp groups, via Facebook, or by putting signs on their caravan doors. A woman explained that people at her site came up with an idea of how to prevent others from coming close to a positive COVID-19 case (Quote 10) and she was encouraged by others to implement it when she had COVID-19. She also told us that if she ran out of household essentials, other residents would get these for her and leave them outside her gate. Her partner added that residents at the side are like “*one big family*” and “*everyone helps each other out*”. Other participants shared similar examples of mutual aid at their sites and in their personal networks to provide food and other necessities during lockdown or self-isolation.

I mean, up here [Traveller site] we had to put signs on our gate […] ‘please don’t come in’ […] because there was this couple over there that had [COVID-19]. And then everybody was like, maybe we should put a sign on your gate so people don’t come in or, like, the postman doesn’t come in.

*Quote 10 (Interview 22)*

Residents helped raise awareness about COVID-19 cases at their sites. They phoned each other and used Facebook to tell others they had COVID-19 so that they would not come to visit (Quote 11, Quote 12). When talking about this type of community-led contact tracing, one participant explained that “*Travellers […] are very good at spreading news*” (Quote 12).

As I say, it’s just, you phone one another, like I say, I’ve got some telephone numbers in there.

*Quote 11 (Interview 26)*

[at the beginning] it was very frightening because it was like black plague, don’t say you’ve got it […] But now I think it’s got better because as soon as someone’s getting it now it’s straight up on social media they say, come back positive, anyone that’s been in contact with me stay away, self test. […] So as soon as someone had it whatever and they put it up [on Facebook] and made it public then everyone would tell everyone or they’d phone one another and say, so and so has got it or your children mixed with your children and this and that so they’d let one another know.

*Quote 12 (Interview 37)*

One participant told us how if someone got COVID-19 at her site, they would “*get a text straight away so then that person's avoided*”. Men in the site would also remind other men with COVID-19 to self-isolate (Quote 13).

“Someone or the lads will probably say to them, dude, can you do me a favour, will you isolate, you can’t come up, like, because there’s so many sick people here”

*Quote 13 (Interview 41)*

Some participants said they did not want their whereabouts to be tracked by the National Health Service (NHS) COVID-19 app and had privacy concerns about the personal data recorded by the app (Quote 14). This mobile phone app tracked user movement, captured proximity between users, notified them when they had been in contact with a positive case, and allowed subsequent digital contact tracing of a positive case when approved by the user (Wymant et al., 2021).

I don’t think the government should be knowing where you’re at, that’s one thing I, I stand by strongly. […] that really scared me, like to think they was going to know where I was and what I was doing, sort of thing. Like why is that important to anything? If I have Covid, I’m going to tell you, or trust that I will tell you.

*Quote 14 (Interview 38)*

Community-led mechanisms also evolved to support others during self-isolation and lockdowns and to help with COVID-19 information. Participants described receiving and providing support from their communities, e.g. helping obtain food, helping others who could not read and write or who did not have access to the internet. A woman told us about a private (non-council) site where the man who ran the site organised shopping for residents and restricted movements on and off the site. (Quote 15):

There was a lot of private sites, not council, err, Gypsy sites. With Gypsy men that run them. They absolutely stopped anybody coming on and off. And he [the man running the site] got the list for shopping or everything off the full site and he went. There was no men allowed off, or women. […] and he [the man running the site] was protecting the people on the site. Stopping from other residents coming round riding on, having a chat. So he took a lot of responsibility away off people what was already on there. […] So basically he did do a lot for the people what was on the site that was vulnerable.

*Quote 15 (Interview 26)*

There was solidarity among residents living on the same site. There was a case where residents risked fines to help each other when there were many COVID-19 cases on the site (Quote 16):

We helped out like, mix and match or whatever, we all used to walk up and down here [in the site] with all masks on, we’d stand outside each other’s gates with masks on, and as soon as we saw like the council coming, we had to run indoors, because you’d get a fine, wouldn’t you, for being outside? Because […] It’s council land, it’s not private land. […] We even asked to be locked down [the council site]. We asked for the gates to be shut, and they refused, to stop anyone coming on here […] Because everyone on here had Covid, we didn’t want like, to like, say you to come on here, or the postman to come on here or anyone to come on here and get Covid […].

*Quote 16 (Interview 27)*

Participants talked about a shared sense of community on the sites and within Gypsy and Traveller communities in general. One participant said: “*Travellers do look out for Travellers*”.

## 5 Discussion

We have shown how Gypsy and Traveller communities supported themselves and took collective action to protect health and contain COVID-19 in the context of chronic and ongoing marginalisation and discrimination, poor healthcare experiences, police harassment, surveillance and tough living conditions. Free access to home-administered virus test kits was crucial to enable communities to exercise agency and demand their right to health.

Communities were highly motivated to protect themselves. Self-led solutions evolved, including community-facilitated testing and community-led contact tracing. Participants developed their own solutions to notify others about positive cases and carefully self-isolated after a positive test. The fact that test kits were free helped participants access testing and notify their own contacts, empowering communities to protect themselves and others. Their self-led solutions to contain the virus were not easy to achieve; they involved resilience, effort, and personal sacrifices (e.g. loss of income during self-isolation).

The biopolitics of COVID-19 containment approaches (Jayasinghe et al., 2021; Sylvia, 2020), which have placed public health responsibility in the individual actions of citizens, contrasted with the broader healthcare neglect, discrimination, poverty and constrained living conditions participants experienced. Disregard of people's voices and health concerns in relation to COVID-19 vaccination were common (Kühlbrandt et al., 2023).

Other studies have found that Gypsy and Traveller communities are concerned about poor health conditions being associated with poor environmental conditions such as those at sites located in polluted areas (Office for National Statistics (ONS), 2022b). We found infrastructure problems on sites, locked gates that blocked access to ambulances, problems getting post (including health services letters), and in some cases no access to running water and electricity. It is not surprising then that under these circumstances, communities resorted to self-reliance and organised themselves to provide mutual support and protect health. Participants wanted to avoid bringing the virus into their homes or communities, and drew on widely available diagnostic testing to support their protective endeavours. Because of their marginalised status, Gypsy and Traveller communities are accustomed to having to care for themselves rather than rely on outside support (Burrows et al., 2021) resorting to self-reliance in multiple dimensions of life, from education and health, to self-employment (Office for National Statistics(ONS) (2022a).

The findings illustrate the paradox intrinsic to the biopolitics of public health containment approaches: individuals are asked to work to protect all “in the name of life and health” (Rabinow & Rose, 2006, p. 195), yet those pushed to live on the margins struggle to claim their right to health. Infection control must be viewed as part of a wider system if it truly is to protect the health of all. We need to consider how interactions with the state care and criminal justice systems, legislation that criminalises certain forms of living, social exclusion, discriminatory treatment from official bodies, and constrained material conditions, can work together as “necropolitical assemblages” (Grenfell et al., 2022), to institute who it is that matters and whose health is deserving of care, versus who is “disposable” (Mbembe, 2003, p. 27) while simultaneously being tasked by the government to take responsibility for the public good.

In contrast to the emphasis on individual responsibility of the ideal “pandemic citizen” (Maunula, 2013) which underlies the biopolitics of COVID-19 public health approach, what our study illustrates is relational forms of “acts of citizenship” (Isin, 2008) galvanised via collective action and community solidarity. Participants in our study talked about a strong sense of community, and it is not clear whether such community-led initiatives and self-reliance on community resources and aid, would have emerged elsewhere. Social networks, strong social ties and solidarity within Gypsy and Traveller communities are perceived by members of these communities as important sources of a sense of wellbeing, particularly in the face of their social exclusion and discrimination from wider society (Smith & Ruston, 2013). From our own lived experience (RS, SF), and discussions with key informants from these communities about the findings presented in this paper, we are aware that in the face of chronic discrimination and marginalisation experienced by Gypsies and Travellers, these communities have had (pre-COVID-19) to turn inwards to care for themselves, seek other informal ways to support themselves and build resilience. Yet, we also know that for some, particularly for illiterate members of the community, lockdown and pandemic regulations meant losing informal sources of support due to difficulties accessing those contacts who one would in the past have approached for help.

While we sought to engage the most diverse range of participants, we do not claim to provide a comprehensive picture of all Gypsy and Traveller community needs and experiences relating to COVID-19. Although the compressed timeframe for this study and concurrent COVID-19 Omicron-variant wave meant that we recruited fewer participants than planned in the north of England, it seems plausible that experiences outside our study sites would be similar to those described here, where a strong sense of community and mutual support mechanisms exist.

Other studies in the general population, also found that support emerged from communities across England during the COVID-19 pandemic to provide practical and essential help to residents (e.g. help with shopping, prescription deliveries) (Ellis, Wilson, McCabe, & Macmillan, 2022; Ward et al., 2022). Stronger community responses tended to be among more cohesive communities and those with stronger relationships with their local authoriries (Ellis et al., 2022). Gypsy and Traveller community action to support themselves during the pandemic might have been strengthened further if they had had better access to resources and support from official sources.

Our study shows how for those living on the margins, exercising their citizenship to claim their right to health in the context of infection emergencies, requires community support and self-reliance on community networks and resources. This is likely to be relevant to other marginalised communities and outside our specific study locations. Free, easy access to rapid home testing was crucial for marginalised communities to take protective action against COVID-19, to empower themselves to exercise their right to health. Yet free access to testing and resources is not enough for individuals to act with agency and self-regulate their own life to manage the risks of the virus, as is assumed in individualistic public health approaches to virus containment.

An enabling policy, material, and social environmment is crucial for people to engage in relational “acts of citizenship” (Isin, 2008) by which they can claim their right to health and demand resources to support themselves in infection prevention and control and also in other areas of health. To improve preparedness for future emergencies, and to redress longstanding health and social inequalities experienced by Gypsy and Traveller communities and other marginalised groups, it is essential that marginalisation is addressed, with healthcare services links into communities built via trusted parties who could act as community liaisons to help meet community needs including illiteracy issues. Lack of formal support and disregard for people's voices (including virus-related concerns) need to be addressed now and in future infection emergencies, to avoid replicating and reinforcing historic and ongoing experiences of marginalisation and racism in healthcare services. Experiences of surveillance, policing and discrimination in official institutions might have contributed to participants' unwillingness to engage with the NHS COVID-19 app. Policies such as the Police, Crime, Sentencing and Courts Bill, which criminalises nomadic lives, and experiences of police harassment, might contribute to individuals' reluctance to provide their personal data, and exclude them from formal support systems and resources. It is important to provide mechanisms that support community-led responses without having to engage with government systems that require personal data if rapid contact tracing and action is needed to prevent spread. Community-led contact notification can be rapid and should be actively supported. Some of the recommendations suggested by members of Gypsy and Traveller communities in our dialogue sessions included: (1) allowing PCR tests without giving personal data, (2) accepting internal community contact tracing and notification mechanisms in lieu of government systems, and (3) allowing people to self-organise to do community contact tracing via civil society organisations so that personal data is not given to outsiders. When trusted by communities, civil society organisations can help find people who are not on official records.

Community-led solutions to infection containment must be at the centre of public health responses. Community action can open up novel infection control strategies, but communities must be supported to do this. Mutual aid strategies and community collaboration developed by and for communities should receive material, political and technical support. This is crucial; Gypsy and Traveller communities have had limited access to support for self-organisation compared with other groups (Cemlyn et al., 2009).

Communities should also be at the core of co-production of pandemic preparedness and response strategies, and wider health services, to ensure these are inclusive and meet their needs (Marston et al., 2020). Institutional structures should be created to support communities’ involvement in co-designing health solutions with and by communities. For example, trusted individuals and civil society organisations who are part of these communities can help to bridge public health official bodies and communities to guide co-production efforts, build networks, and co-design effective, efficient, and acceptable strategies for better future emergency preparedness and response.

## Author contributions

Alicia Renedo: Conceptualisation (ideas; formulation or evolution of overarching research goals and aims); Data curation; Formal analysis; Funding acquisition; Investigation; Methodology; Project administration; Resources; Supervision; Writing - original draft; Writing - review & editing.

Rachel Stuart: Conceptualisation (ideas); Data curation; Formal analysis; Investigation; Writing - original draft, Writing - review & editing.

Charlotte Kühlbrandt: Conceptualisation (ideas); Data curation; Formal analysis; Investigation; Writing - review & editing.

Serena Farrow : Investigation; Analysis interpretation; Writing - review & editing.

Pippa Grenfell: Conceptualisation (ideas); Data curation; Formal analysis; Investigation; Writing - review & editing.

Catherine McGowan: Conceptualisation (ideas); Data curation; Formal analysis; Investigation; Writing - review & editing.

Sam Miles: Conceptualisation (ideas); Data curation; Formal analysis; Investigation; Writing - review & editing.

Cicely Marston: Conceptualisation (ideas; formulation or evolution of overarching research goals and aims); Data curation; Formal analysis; Funding acquisition; Investigation; Methodology; Project administration; Resources; Supervision; Writing - original draft, Writing - review & editing.

## Note about co-authorship

Author order is difficult to assign in a complex project with multiple contributions and for this reason authors 4-6 are listed in alphabetical order by surname. We consider their input to be equally valuable to this paper.

## Funding

This paper is independent research commissioned and funded by the National Institute for Health Research (NIHR) Policy Research Programme. The views expressed in this publication are those of the authors and not necessarily those of the NHS, the NIHR, the Department of Health and Social Care or its arm’s length bodies, and other Government Departments.

## Declaration of competing interest

The authors declare that they have no known competing financial interests or personal relationships that could have appeared to influence the work reported in this paper.
